# Comparison of Promoter Hypermethylation Pattern in Salivary Rinses Collected with and without an Exfoliating Brush from Patients with HNSCC

**DOI:** 10.1371/journal.pone.0033642

**Published:** 2012-03-16

**Authors:** Wenyue Sun, David Zaboli, Yan Liu, Demetri Arnaoutakis, Tanbir Khan, Hao Wang, Wayne Koch, Zubair Khan, Joseph A. Califano

**Affiliations:** 1 Department of Otolaryngology-Head and Neck Surgery, Department of Oncology, The Sidney Kimmel Comprehensive Cancer Center at Johns Hopkins, Johns Hopkins Medical Institutions, Baltimore, Maryland, United States of America; 2 Department of Surgery, Department of Oncology, The Sidney Kimmel Comprehensive Cancer Center at Johns Hopkins, Johns Hopkins Medical Institutions, Baltimore, Maryland, United States of America; 3 Division of Oncology Biostatistics, Department of Oncology, The Sidney Kimmel Comprehensive Cancer Center at Johns Hopkins, Johns Hopkins Medical Institutions, Baltimore, Maryland, United States of America; 4 Milton J. Dance Head and Neck Center, Greater Baltimore Medical Center, Baltimore, Maryland, United States of America; Dartmouth College, United States of America

## Abstract

**Background:**

Salivary rinses have been recently proposed as a valuable resource for the development of epigenetic biomarkers for detection and monitoring of head and neck squamous cell carcinoma (HNSCC). Both salivary rinses collected with and without an exfoliating brush from patients with HNSCC are used in detection of promoter hypermethylation, yet their correlation of promoter hypermethylation has not been evaluated. This study was to evaluate the concordance of promoter hypermethylation between salivary rinses collected with and without an exfoliating brush from patients with HNSCC.

**Methodolgy:**

57 paired salivary rinses collected with or without an exfoliating brush from identical HNSCC patients were evaluated for promoter hypermethylation status using Quantitative Methylation-Specific PCR. Target tumor suppressor gene promoter regions were selected based on our previous studies describing a panel for HNSCC screening and surveillance, including *P16*, *CCNA1*, *DCC*, *TIMP3*, *MGMT*, *DAPK* and *MINT31*.

**Principal Findings:**

In salivary rinses collected with and without brush, frequent methylation was detected in *P16* (8.8% vs. 5.2%), *CCNA1* (26.3% vs. 22.8%), *DCC* (33.3% vs. 29.8%), *TIMP3* (31.6% vs. 36.8%), *MGMT* (29.8% vs. 38.6%), *DAPK* (14.0% vs. 19.2%), and *MINT31* (10.5% vs. 8.8%). Spearman's rank correlation coefficient showed a positive correlation between salivary rinses collected with and without brush for *P16* (ρ = 0.79), *CCNA1* (ρ = 0.61), *DCC* (ρ = 0.58), *TIMP3* (ρ = 0.10), *MGMT* (ρ = 0.70), *DAPK* (ρ = 0.51) and *MINT31* (ρ = 0.72) (P<0.01). The percent agreement of promoter methylation between salivary rinses with brush and without brush were 96.5% for *P16*, 82.5% for *CCNA1*, 78.9% for *DCC*, 59.7% for *TIMP3*, 84.2% for *MGMT*, 84.2% for *DAPK*, and 94.7% for *MINT31*.

**Conclusions:**

Our study demonstrated strong correlations of gene promoter hypermethylation between salivary rinses collected with and without an exfoliating brush. Salivary rinse collection without using an exfoliating brush may offer a cost effective, rapid, non-invasive, and reliable means for development of epigenetic salivary rinse biomarkers.

## Introduction

Head and neck squamous cell carcinoma (HNSCC) is the sixth most common cancer in the world. More than 40,000 new cases of HNSCC are diagnosed in the United States each year, with a mortality rate of 12,000 U.S. deaths annually. The etiology of HNSCC includes well-known risk factors, such as tobacco smoking and alcohol drinking, as well as infection of human papillomavirus (HPV) [1], [2]. Despite significant improvements in therapeutic modalities, 5-year survival rates are still among the lowest of the major cancers, with loco-regional relapse being the primary cause of death.

Saliva is a readily obtained body fluid that contains cells shed from the mucosal lining of the mouth and throat. It is becoming a promising diagnostic tool for non-invasive and cost effective HNSCC detection. As a type of body fluid, saliva can potentially carry whole cells as well as protein, RNA, and DNA species that allow for detection of cellular alterations related to HNSCC. Salivary proteome analyses for oral cancer has been reported and a number of specific genes and proteins have been proposed as biomarkers for clinical diagnosis, including p53, Cyfra21-1 tissue polypeptide antigen and CD44 [3], [4], [5], [6], [7]. Studies have demonstrated that thousands of cell-free mRNAs and miRNAs are present in saliva and demonstrated the feasibility of using salivary mRNAs/miRNAs for detection of oral cancer [8], [9], [10]. The inactivation of tumor suppressor genes caused by epigenetic changes such as promoter region CpG island hypermethylation has been well established in the literature. Aberrant promoter hypermethylation of cancer-associated genes are common in many human cancers including HNSCC [11]. The detection of gene promoter hypermethylation in salivary rinses has been explored as a potential for diagnostic and monitoring of HNSCC [12], [13], [14], [15]. We have previously published results of salivary rinse screening using promoter hypermethylation-based markers in patients with previously diagnosed HNSCC. Our group has developed a panel for detection of HNSCC by evaluation of salivary rinses from these patients. For the initial screening of 21 genes for salivary rinses, ultimately seven genes (*P16*, *CCNA1*, *DCC*, *TIMP3*, *MGMT*, *DAPK*, and *MINT31*) were selected as part of a panel to distinguish salivary rinses from HNSCC patients and healthy controls [16]. Moreover, we found detection of hypermethylation in pre-treatment salivary rinse DNA appears to be predictive of local recurrence and overall survival [17].

Clinically, salivary rinses can be obtained by brushing oral cavity and oropharyngeal surfaces with an exfoliating brush followed by rinse and gargle. The tissue collected using this technique includes exfoliated epithelial cells from the upper aerodigestive tract, and an exfoliating brush is used to include cells from deep epithelial layers in the oral cavity and oropharynx [12], [16]. This technique allows for a broad sampling of epithelial cells from multiple sites in the upper aerodigestive tract. Alternatively, salivary rinses can be obtained without using an exfoliating brush [18]. To date, salivary rinses collected with and without an exfoliating brush from patients with HNSCC have been widely used in the development of epigenetic biomarkers. However, the correlation of gene promoter hypermethylation between salivary rinses collected with and without an exfoliating brush has not yet been studied.

The ability to detect molecular alterations in salivary rinses has been proposed as a potential low-cost method to detect individuals at risk for head and neck cancer development, and as a potential surveillance tool for HNSC patients. However, use of an exfoliating brush to obtain material in salivary rinses requires administration by a trained health care provider, as well as use of specific, exfoliating brushes with potential added expense. The demonstration that adequate harvest of aberrant, methylated DNA can occur with a less intensive technique that may potentially be performed independently by patients without the need for specialized equipment would make salivary based detection more easily adoptable and more cost effective. In this study, we compared the patterns of promoter hypermethylation in salivary rinses collected with and without brush from patients with HNSCC. We show that salivary rinses collected with and without brush share similar hypermethylation patterns, suggesting that brush use may not be necessary to harvest salivary rinses for the study of DNA hypermethylation in patients with HNSCC.

## Materials and Methods

### Subjects

All human oral salivary rinse samples were obtained and used according to the policies of the Johns Hopkins Medical Institutions. The experimental protocol was approved by the Johns Hopkins Medical Institutions Review Board. Witten informed consent was obtained from each subject prior to the use of their tissue for scientific research. Between June 2005 and Oct 2010, the salivary rinse samples were prospectively collected with an exfoliated brush from patients (n = 197) presenting a previously untreated squamous cell carcinoma from the oral cavity, larynx, or pharynx, at the Department of Otolaryngology-Head and Neck Surgery, Johns Hopkins Medical Institutions (Baltimore, MD). Among the 197 patients, 57 paired salivary rinse samples were also collected without using an exfoliating brush. In our salivary rinse collection, no selection criteria were applied on patients. To obtain clinical information, we reviewed medical records to identify patients with pathologically confirmed HNSCC. Enrollment included collection of demographic information and risk factor history (tobacco and alcohol). Smoking was defined as use of tobacco, chewable or smoked, for at least 1 year continuously. Alcohol use was defined as intake of more than two alcoholic drinks per day.

### Collection of salivary rinse samples

Salivary rinses were obtained from all subjects as previously described [16], [18], [19]. Pretreatment salivary rinse samples were collected before tumor resection on the day of tumor resection. Patients firstly contributed a pretreatment oral rinse by swishing and gargling for 15 s with 20 ml of normal saline solution followed by expectoration (salivary rinse collected without brush). Then an exfoliating brush was used to brush oral cavity and oropharyngeal surfaces followed by rinse and gargling with 20 ml normal saline solution (salivary rinse collected with brush). The tumor site was intentionally avoided during brushing. This technique allows for a broad sampling of epithelial cells from multiple sites in the upper aerodigestive tract. The brush was gently agitated to release the obtained material into saline. After centrifugation, the supernatant was discarded and DNA was isolated from the pellet. The method with brush obviously generates more DNAs than the method without brush.

### DNA extraction

DNA obtained from salivary rinse samples was extracted by the tissue bank by digestion with 50 µg/mL proteinase K (Boehringer) in the presence of 1% SDS at 48°C overnight followed by phenol/chloroform extraction and ethanol precipitation.

### Bisulfite treatment

The DNA obtained from the salivary rinse samples was subjected to bisulfite treatment, using the EpiTect Bisulfite kit from Qiagen according to the manufacturer's conditions, http://www.Qiagen.com. Bifulfite-treated DNA was eluted in 30 µL of elution buffer and stored at −80°C [17], [20].

### Quantitative methylation-specific PCR

The bisulfite-treated DNA was used as a template for fluorescence-based real-time Q-MSP as described previously [21]. The *P16*, *CCNA1*, *DCC*, *TIMP3*, *MGMT*, *DAPK*, *MINT31* and ACTB genes had been previously detected on a prior screen of salivary rinses in HNSCC patients [16], [17]. We had optimized the primer and probe sequences for Q-MSP, and their sequences are published previously [16]. The ratios between the values of the gene of interest and the reference gene ACTB were obtained by TaqMan analysis and used as a measure for representing the relative quantity of methylation in a particular sample (value for gene of interest/value for ACTB gene×100). Fluorogenic PCRs were carried out in a reaction volume of 10 µL of 200 nmol/L of each primer, 100 nmol/L of probe, 0.375 unites of platinum Taq Polymerase (Invitrogen), 100 µmol/L of ROX Reference Dye (Invitrogen), 8.4 mmol/L ammonium sulfate, 33.5 mmol/L Trizma (Sigma), 3.35 mmol/L magnesium chloride, 5 mmol/L mercaptoethanol, and 0.05% DMSO. Each real-time Q-MSP reaction consisted of 1.5 µL of treated DNA solution. Amplifications were carried out in 384-well plates in a 7900 Sequence Detector System (Perkin-Elmer Applied Biosystems). Thermal cycling was initiated with a first denaturation step at 95°C for 2 minutes followed by 50 cycles of 95°C for 15 seconds and 60°C for 1 minute. Each reaction was done in triplicate; the average of the triplicate was considered for analysis. The triplicate reactions also provided evidence of reproducibility of the individual reactions. Standardization was done by collecting leukocytes from a healthy individual and subjecting the cells to methylation in vitro with excess SssI methyltransferase (New England Biolabs) to generate completely methylated DNA, and serial dilutions (45-0.0045 ng) of this DNA were used to construct a calibration curve for each plate [22]. The DNA was then bisulfite treated as described above. Serial dilutions of the DNA were used for constructing the calibration curves on each plate. A separate sample of leukocytes from a healthy individual was obtained and only bisulfite treatment was done on the samples. These samples were used as a negative control for the reactions. There were also several control wells in each plate that contained only the reaction mix and water to ensure that there was no contamination. The results of Q-MSP were analyzed by considering the quantity of mehylation normalized by ACTB as well as the quantity of methylation as a binary event, in which any quantity of methylation in a sample would be considered positive for methyaltion.

### Target gene selection

Genes selected for this study came from a study to develop a panel for HNSCC detection and surveillance in body fluids [16], [17]. These genes included *P16*, *CCNA1*, *DCC*, *TIMP3*, *MGMT*, *DAPK*, and *MINT31*.

### HPV analysis

The HPV status was determined as described previously [17], [23]. In brief, specific primers and probes have been designed to amplify the E6, E7 regions of HPV16. Their sequences are available in previous publications [17], [23]. All the samples were run in duplicate. Primers and probes to a house keeping gene (β-actin) were run in duplicate and parallel to normalize input DNA. Samples in which two results were not concordant were repeated twice in duplicate and were usually due to failed PCR in one of the initial reactions. Each reaction was run for 50 cycles. By using serial dilutions, standard curves were developed for the HPV 16 viral copy number using CaSki (American Type Culture Collection, Manassas, VA) cell line genomic DNA, known to have 600 copies/genome (6.6 pg of DNA/genome). Standard curves were developed for HPV16 E6 and E7, using serial dilutions of DNA extracted from CaSki cells with 50,000 pg, 5,000 pg, 500 pg, 50 pg and 5 pg of DNA. Standard curves were developed as well for the β-actin housekeeping gene (2 copies/genome), using the same serial dilutions of the CaSki genomic DNA. This additional step allowed for relative quantification of the input DNA level and final quantity as the number of viral copies/genome/cell. HPV copy number >0.1 copy/cell for tumor samples were regarded as positive. For saliva samples, any amplified sample with HPV E6 or E7 amplification at control β-actin amplification of 10 ng was regarded as positive.

### Statistical analysis

The promoter methylation of seven individual genes was analyzed in two ways: as a continuous variable and as a binary variable (methylation versus no methylation) by dichotomizing each gene at zero. As a continuous variable, levels of promoter methylation were summarized with scatter plots. For each of the seven genes, the correlation of promoter methylation between salivary rinses collected with and without an exfoliating brush was determined by calculating a Spearman's correlation coefficient. As a binary variable (methylation versus no methylation), the frequency of methylated and unmethylated cases for seven individual genes were determined. For each of these genes, we evaluate the agreement between the methods with and without brush as the proportion of samples that is classified to the same methylation status by them. Concordance was assessed by using Cohen's kappa (κ), a coefficient of agreement that corrects for chance [24]. Landis and Koch proposed categories for judging κ values: κ less than 0.00 was poor, 0.00 to 0.20 was slight, 0.21 to 0.40 was fair, 0.41 to 0.60 was moderate, 0.61 to 0.80 was substantial, and 0.81 to 1.00 was almost perfect [25]. Association between methylation status of each studied gene and clinical and pathologic variables in salivary rinse collected with or without an exfoliating brush were analyzed by multivariate analysis using logistic regression models. All statistical analyses were performed in SAS (Cary, NC). All statistical tests were two sided. A p value less than 0.05 would indicate statistical significance.

## Results

### 1. Clinical and pathologic characteristics

Fifty-seven patients were included in this study (Table 1). HNSCC Patients were mainly males (77.2%), Caucasians (93.0%) with ages ranging from 29 to 87 years old (median, 56.5 years). Alcohol or tobacco consumption (current or former) was reported by 59.7% and 70.2%, respectively. HPV status was positive in 26 cases (45.6%). Primary tumor sites included: oral cavity, 22 cases (38.6%); oropharynx, 30 (52.6%) and other, 5 (8.8%). Pathological clinical stage at diagnosis was pT1/pT2 in 43 cases (75.5%), pT3/pT4 in 11 (19.3%) and pTx in 3 (5.3%); pN0 in 16 cases (28.1%) and pN+ in 41 (71.9%). With regard to clinical TNM classification, 13 patients had stage I/II and 44 patients, stage III/IV. This study intends to evaluate the relationship of the methods of salivary rinse collection with and with exfoliating brush to determine promoter hypermethylation in salivary rinses, therefore we do not include patient outcomes. In this respect, for salivary rinse samples collected with an exfoliating brush, we recently published a prognostic analysis of these salivary rinse methylation biomarkers [26].

**Table 1:** Baseline Characteristics of the Patients.

Characteristic	No. of Patients	%
Age at study entry		
<55 yr	23	40.4%
55–64 yr	17	29.8%
>64 yr	17	29.8%
Mean(Year)		56.5
Range		29–87
Sex		
Male	44	77.2%
Female	13	22.8%
Race		
Caucasian	53	93.0%
African	3	5.3%
Asian	1	1.8%
Smoking status		
Never Smoked	17	29.8%
Former	20	35.1%
Current	14	24.6%
Unknown	6	10.5%
Alcohol		
Never Used	10	17.5%
Used	40	70.2%
Unknown	7	12.3%
HPV		
Negative	31	54.4%
Positive	26	45.6%
Primary Site		
Oral cavity	22	38.6%
Oropharynx	30	52.6%
Other	5	8.8%
Pathological tumor Stage		
T1	27	47.4%
T2	16	28.1%
T3	5	8.8%
T4	6	10.5%
Tx	3	5.3%
Pathological nodal stage		
N0	16	28.1%
N1	7	12.3%
N2	34	59.6%
Clinical TNM stage		
I	10	17.5%
II	3	5.3%
III	7	12.3%
IV	37	64.9%

### 2. Frequencies of promoter hypermethylation in salivary rinses collected with and without an exfoliating brush from 57 patients with HNSCC

**Figure 1:**
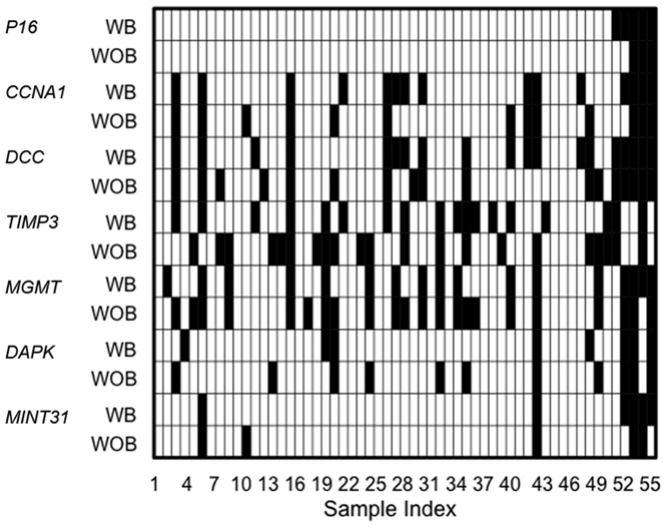
Aberrant promoter methylation in the DNAs from salivary rinses collected with or without an exfoliating brush from 57 HNSCC cancer patients. Each column represents a patient, and each row the methylation status of the given gene in salivary samples collected with brush or without brush. Black shading indicates promoter hypermethylation and white indicates lack of promoter methylation. WB, salivary rinses collected with an exfoliating brush; WOB, salivary rinses collected without brush.

We tested promoter methylation pattern of *P16*, *CCNA1*, *DCC*, *TIMP3*, *MGMT*, *DAPK*, and *MINT31* in 57 paired salivary rinses that were collected with or without an exfoliating brush from the above patients with HNSCC. These seven genes selected for this study were from our previous studies to develop a panel for HNSCC detection and surveillance in salivary rinses. The methylation levels of these selected genes in salivary rinses collected with or without an exfoliating brush from HNSCC patients were shown in Figure S1. In the salivary rinse samples collected with an exfoliating brush, frequent methylation was detected in *P16* (8.8%), *CCNA1* (26.3%), *DCC* (33.3%), *TIMP3* (31.6%), *MGMT* (29.8%), *DAPK* (14.0%), and *MINT31* (10.5%) (Table 2). In the salivary rinse samples collected without brush, the methylation frequencies observed were 5.2% for *P16*, 22.8% for *CCNA1*, 29.8% for *DCC*, 36.8% for *TIMP3*, 38.6% for *MGMT*, 19.2% for *DAPK*, and 8.8% for *MINT31* (Table 2). Figure 1 summarized the methylation profiles of each of the seven genes for the 57 pairs of salivary rinses collected with brush and without brush from patient with HNSCC. Methylation genes had been categorized as methylated for any value greater than zero.

**Table 2:** Frequency of promoter methylation of the seven genes analyzed in the salivary rinses collected with or without an exfoliating brush from 57 HNSCC patients, and Spearman correlation of promoter methylation levels.

Gene	Methylation Positive %(no. of methylation positive/no. total cases)		Spearman's Correlation	
	WB§	WOB‡	Coefficient	P value
*P16*	8.8 (5/57)	5.2 (3/57)	0.79	<0.0001
*CCNA1*	26.3 (15/57)	22.8 (13/57)	0.61	<0.0001
*DCC*	33.3 (19/57)	29.8 (17/57)	0.58	<0.0001
*TIMP3*	31.6 (18/57)	36.8 (21/57)	0.10	0.49
*MGMT*	29.8 (17/57)	38.6 (22/57)	0.70	<0.0001
*DAPK*	14.0 (8/57)	19.2 (11/57)	0.51	<0.0001
*MINT31*	10.5 (6/57)	8.8 (7/57)	0.72	<0.001

§ WB = salivary rinses collected with an exfoliating brush.

‡ WOB = salivary rinses collected without a brush.

### 3. Concordance of promoter methylation of the seven individual genes in salivary rinses collected with and without an exfoliating brush from 57 patients with HNSCC

We analyzed the correlation of promoter methylation status of these seven individual genes between salivary rinses collected with brush and without brush by Spearman correlation analysis, using methylation levels as a continuous variable (Table 2). We found strong correlations between the salivary rinses with brush and without brush for *P16* (ρ = 0.79, P<0.0001), *CCNA1* (ρ = 0.61, P<0.0001), *DCC* (ρ = 0.58, P<0.0001), *MGMT* (ρ = 0.70, P<0.0001), *DAPK* (ρ = 0.51, P<0.0001) and *MINT31* (ρ = 0.72, P<0.001).

We also assessed concordance of the promoter methylation status of these seven individual genes between salivary rinses collected with and without an exfoliating brush using methylation levels as a categorical variable. As shown in Table 3, the percent agreement between the salivary rinses with brush and without brush was 96.5% for *P16*, 82.5% for *CCNA1*, 78.9% for *DCC*, 59.7% for *TIMP3*, 84.2% for *MGMT*, 84.2% for *DAPK*, and 94.7% for *MINT31* (Table 3). Furthermore, we used Cohen's kappa (κ) to evaluate the concordance of promoter methylation between salivary rinses collected with and without an exfoliating brush. Of note, the kappa statistic depends on the underlying methylation prevalence, which may lead to smaller kappa even when the % agreement is higher (Table 3). Overall, moderate agreements of promoter methylation at *CCNA1* (κ = 0.53), *DCC* (κ = 0.51), and *DAPK* (κ = 0.43) were noted between salivary rinse with brush and without brush; substantial agreements of promoter methylation at *P16* (κ = 0.73), *MGMT* (κ = 0.65), and *MINT31* (κ = 0.70) were demonstrated between salivary rinses with brush and without brush (Table 3).

**Table 3:** Concordance between salivary rinses collected with or without an exfoliating brush from 57 HNSCC patients.

			Agreement	Kappa	95% CI§§
Genes	WOB‡ (−)	WOB(+)	(%)		
*P16*					
WB§ (−)	52	0	96.5	0.73	0.38–1
WB (+)	2	3			
*CCNA1*					
WB (−)	38	4	82.5	0.53	0.27–78
WB (+)	6	9			
*DCC*					
WB (−)	33	5	78.9	0.51	0.27–0.75
WB (+)	7	12			
*TIMP3*					
WB (−)	26	13	59.7	0.11	−0.16–0.37
WB (+)	10	8			
*MGMT*					
WB (−)	33	7	84.2	0.65	0.45–0.86
WB (+)	2	15			
*DAPK*					
WB (−)	43	6	84.2	0.43	0.13–0.74
WB (+)	3	5			
*MINT31*					
WB (−)	50	1	94.7	0.70	0.38–1
WB (+)	2	14			

§ WB = salivary rinses collected with an exfoliating brush.

‡ WOB = salivary rinses collected without a brush.

§§ CI = Confidence Interval.

### 4. Association of promoter methylation in salivary rinses collected with brush or without brush with clinical and pathologic characteristics

We determined the association of the promoter methylation of each marker individually with the clinical and pathological variables in HNSCC using multivariate logistic regression. The clinical and pathological variables considered in the multivariate analysis were age, gender, smoking status, HPV status, primary tumor site, pathological tumor stage, pathological nodal stage and clinical TNM stage. In the salivary rinse samples collected with brush, smoking status was associated with promoter methylation of *TIMP3*; HPV status was associated with promoter methylation of *CCNA1*, *DCC*, and *MGMT*; primary tumor site (Oral cavity) was associated with promoter methylation of *CCNA1*, *DCC* and *DAPK*; clinical TNM stage were associated with promoter methylation of *P16*, *DCC*, and *MINT31*; and pathological nodal stage was associated with promoter methylation of *P16* and *MINT31* (Table S1). In the salivary rinse samples collected without brush, primary tumor site was associated promoter methylation of *CCNA1*; and clinical TNM stage was associated with promoter methylation of *CCNA1* and *DCC* (Table S1). Due to our sample size, the association between clinical and pathological characteristics and gene promoter methylation should be interpreted with caution.

## Discussion

Body fluids such as saliva are potential resources for development of biomarkers for detection, diagnosis, and prognosis of HNSCC. Aberrant promoter hypermethylation has been recently proposed as a means for detection of HNSCC in salivary rinses. Recently, our group published the utility of evaluating the promoter region methylation status of various genes as a tool for detection of HNSCC. In our study, seven genes, comprised of *DAPK*, *DCC*, *MINT31*, *TIMP3*, *P16*, *MGMT* and *CCNA1*, were identified as part of a panel that could distinguish salivary rinses from HNSCC patients and healthy controls [16]. With a pilot cohort of 61 HNSCC patients, we also found that the detection of these markers in pretreatment salivary rinse is a likely prognostic indicator for local recurrence and poor survival [17].

Salivary rinses used for promoter hypermethylation assay in the literature have been collected either with or without an exfoliating brush [16], [18]. An exfoliating brush could be used to include cells from deep epithelial layers in the oral cavity and oropharynx. It also allows for a broad sampling of epithelial cells from multiple sites in the upper aerodigestive tract. Although both salivary rinses collected with and without an exfoliating brush has been reported in detection of promoter hypermethylation, the clinical significance of exfoliating brush use in salivary rinse collection for detection of promoter hypermethylation is unknown. There has been no direct study of the correlation of promoter hypermethylation between salivary rinses collected with and without an exfoliating brush.

In this study, we first determined the promoter methylation pattern of seven individual genes, including *P16*, *CCNA1*, *DCC*, *TIMP3*, *MGMT*, *DAPK*, and *MINT31*, in 57 paired salivary rinses collected with or without an exfoliating brush from patients with HNSCC, and then evaluated the concordance of promoter hypermethylation between salivary rinses collected without brush and those with brush. As shown in Table 1, the clinical and pathological characteristics of these 57 patients with HNSCC appeared comparable to the patient cohort we previously published, and were broadly representative of standard clinical practice. To circumvent the possible confounding factors that may be involved in salivary rinse collection, we have collected each pair of salivary rinses (with and without an exfoliating brush) sequentially during one visit to the physician's office. Quantitative methylation-specific PCR was used to detect the promoter hymermethylation in salivary rinse sample DNA. This real-time PCR methodology allows a more objective, robust, and rapid assessment of promoter methylation status. Give the sensitivity of the QMSP technique used to detect the presence of methylated alleles in a background of normal at a threshold of 1/1,000 to 1/10,000, this strategy allowed us to define methylated genes that were highly specific for tumor, and rarely or never present in any of the aerodigestive sites that shed cells in salivary rinses.

We reported that promoter hypermethylation frequencies of *P16*, *CCNA1*, *DCC*, *MGMT*, *DAPK*, and *MINT31* could be detected in salivary rinses collected without an exfoliating brush at levels comparable to those in salivary rinses collected with brush. We showed that the promoter hypermethylation frequencies of these studied genes in salivary rinses collected with and without an exfoliating brush were between 8.8% and 31.6% and between 5.2% and 38.6%, respectively [16], [17].

Our study also demonstrated a concordance of gene promoter methylation between salivary rinses collected with an exfoliating brush and without brush from patients with HNSCC. In our study, Spearman rank analysis showed a strong correlation for promoter hypermethylation of *P16*, *CCNA1*, *DCC*, *MGMT*, *DAPK*, and *MINT31*, although a weak correlation for *TIMP3* promoter hypermethylation was found (The reason why *TIMP3* was poorly concordant was unknown.). We also found a strong agreement for promoter hypermethylation of these markers (Table 3). Meanwhile, as revealed by Cohen's kappa statistic, we found moderate agreements of promoter methylation at *CCNA1*, *DCC* and *DAPK* and substantial agreements of promoter methylation at *P16*, *MGMT* and *MINT31*. It should be noted that the kappa statistic also depends on the underlying methylation prevalence that may lead may lead to smaller kappa even the percent agreement is higher. In addition, the techniques we used for paired salivary rinse collection may attenuate the concordances of gene promoter methylation to some extent, making it seem like the techniques are less agreeable than they actually are. We don't exclude the possibility that the initial salivary rinses without brushing capture much of the loose epithelial and tumor cells whereas the rinses with brushing capture fewer tumor cells, since there was already a prior rinse. As an additional point, it remain to be investigated whether detection of methylation markers from saliva more than once will increase the total percentage of the positive cases, no matter using brush or not.

The concordance of promoter hypermethylation between salivary rinses collected with and without an exfoliating brush may have biological implications. To date, the mechanism leading to the presence of gene promoter hypermethylation in salivary rinse is not well understood. It is likely that 1) aggressive tumors may undergo increase rate of mechanical dissociation or shedding into salivary rinses. Those tumor with a higher burden of epigenetic alteration would be more frequently detected in salivary rinses; 2) salivary rinse tumor DNA with epigenetic alterations may also originate from cells that have left the primary site and have invaded the circulatory system but are still not capable of metastasis to new organ; 3) premalignant clonal patches expanded will beyond primary tumor location, resulting a large surface area of epigenetically altered cells to shed into the saliva [17], [19]. Previous studies hypothesized that salivary rinses collected without an exfoliating brush may not have enough oropharyngeal cells to meet cutoffs for positive biomarker findings in case. Brushing, which are site specific, may help overcome these obstacles [27]. However, based on our current study, at least for study of gene promoter methylation in salivary rinses, detection of gene promoter hypermethylation between salivary rinses collected with and without an exfoliating brush is concordant, suggesting that an exfoliating brush is not necessary to be used for salivary rinse collection.

The concordance of promoter hypermethylation between salivary rinses collected with and without an exfoliating brush have potentially important clinical implications. In comparison to collection of salivary rinses with an exfoliating brush for development of epigenetic biomarkers for epigenetic study, there are numerous potential advantages for the collection of salivary rinses without using an exfoliating brush. For instance, the method is non-invasive (the sample is relatively easy and painless to acquire), it has the potential for low cost (no expenses of exfoliating brush), and lends itself to easy administration (it is likely patients can perform this collection by themselves). Thus, the collection of salivary rinses without using an exfoliating brush could be an efficient, cost effective and reliable method for obtaining material for detection of HNSCC related markers.

In summary, the present study compared the detection of similar promoter hypermethylation frequencies of seven individual genes between salivary rinses collected with and without an exfoliating brush from patients with HNSCC. Moreover, our study for the first time demonstrated a strong concordance of gene promoter hypermethylation between salivary rinses collected with and without brush. This study suggests that use of an exfoliating brush may not be necessary for salivary rinse collection for the detection of promoter hypermethylation biomarkers of HNSCC detection.

## Supporting Information

Figure S1 **Promoter methylation levels for seven genes (** ***P16*** **,** ***CCNA1*** **,** ***DCC*** **,** ***TIMP3*** **,** ***MGMT*** **,** ***DAPK*** **and** ***MINT31*** **) in the DNAs from salivary rinses collected with and without an exfoliating brush from 57 HNSCC cancer patients.** The quantity of methylated allele of each gene was expressed as the ratio of the amount of polymerase chain reaction products amplified from the methylated gene to the amount amplified from the reference gene β actin multiplied by 100.
(TIF)

Table S1 **Association of each marker in salivary rinses collected with or without brush with selected features.** Association between methylation status of each studied gene and clinical and pathologic variable in 57 salivary rinses collected with or without an exfoliating bursh were analysed by multivariate analysis using logistic regression models. Odds Ratios and 95% Confidence Interval was shown in the table. Statistical Significance was indicated as red.
(DOCX)
