# Supplementary figures and images for: Comparison of Promoter Hypermethylation Pattern in Salivary Rinses Collected with and without an Exfoliating Brush from Patients with HNSCC
Source: PLoS One. 2012 Mar 16;7(3):e33642. doi: 10.1371/journal.pone.0033642 (PMC3306276; doi:10.1371/journal.pone.0033642)

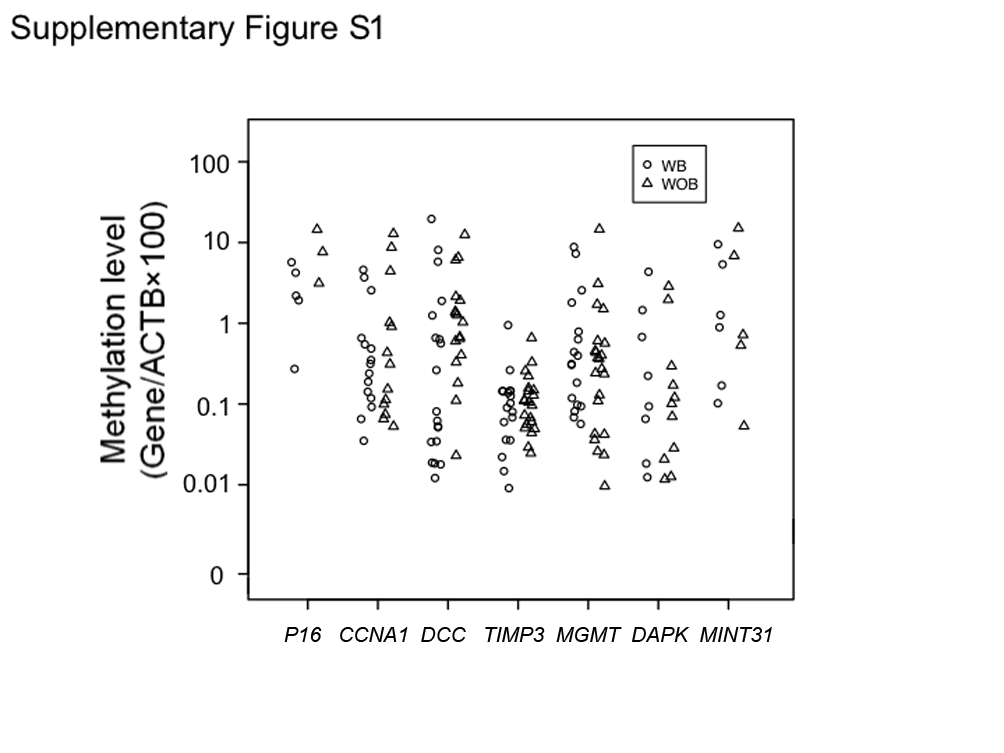

Supplement: Figure S1 — Promoter methylation levels for seven genes ( P16 , CCNA1 , DCC , TIMP3 , MGMT , DAPK and MINT31 ) in the DNAs from salivary rinses collected with and without an exfoliating brush from 57 HNSCC cancer patients. The quantity of methylated allele of each gene was expressed as the ratio of the amount of polymerase chain reaction products amplified from the methylated gene to the amount amplified from the reference gene β actin multiplied by 100. (TIF) [file pone.0033642.s001.tif]
