# Supplementary material for: Comparison of Promoter Hypermethylation Pattern in Salivary Rinses Collected with and without an Exfoliating Brush from Patients with HNSCC
Source: PLoS One. 2012 Mar 16;7(3):e33642. doi: 10.1371/journal.pone.0033642 (PMC3306276; doi:10.1371/journal.pone.0033642)
Supplement: Table S1 — Association of each marker in salivary rinses collected with or without brush with selected features. Association between methylation status of each studied gene and clinical and pathologic variable in 57 salivary rinses collected with or without an exfoliating bursh were analysed by multivariate analysis using logistic regression models. Odds Ratios and 95% Confidence Interval was shown in the table. Statistical Significance was indicated as red. (DOCX) [file pone.0033642.s002.docx]

**Supplementary Table S1. Association of each marker in salivary rinses collected with or without brush with selected clinical features.**

|  |  |  |  |  |  | Odds | Ratio | (95% | Ci) |  |  |  |  |  |
| --- | --- | --- | --- | --- | --- | --- | --- | --- | --- | --- | --- | --- | --- | --- |
|  | *P16* |  | *CCNA1* |  | *DCC* |  | *TIMP3* |  | *MGMT* |  | *DAPK* |  | *MINT31* |  |
|  | WB§ | WOB‡ | WB | WOB | WB | WOB | WB | WOB | WB | WOB | WB | WOB | WB | WOB |
|  |  |  |  |  |  |  |  |  |  |  |  |  |  |  |
| Smoking Status | 2.13 [0.22, 20.72] | 1.06 [0.09, 12.4] | 0.74 [0.2, 2.74] | 0.9 [0.23, 3.55] | 1.77 [0.47, 6.66] | 0.72 [0.22, 2.44] | 0.27 [0.08, 0.94] | 0.47 [0.14, 1.49] | 1.55 [0.41, 5.88] | 1.52 [0.48, 4.84] | 2.76 [0.3, 25.68] | 1.28 [0.29, 5.6] | 1  [0.16, 6.09] | 0.77 [0.12, 5.04] |
| HPV | NA | NA | 0.16 [0.04, 0.61] | 0.31 [0.09, 1.08] | 0.27 [0.08, 0.86] | 0.47 [0.16, 1.42] | 0.87 [0.28, 2.68] | 0.96 [0.33, 2.76] | 0.26 [0.08, 0.87] | 0.7 [0.25, 1.96] | 0.69 [0.15, 3.09] | 0.55 [0.15, 2.02] | NA | 0.16 [0.02, 1.52] |
| Primary Tumor Site¦ | NA | NA | 0.14 [0.03, 0.57] | 0.09 [0.02, 0.43] | 0.12 [0.03, 0.45] | 0.41 [0.13, 1.25] | 0.86 [0.28, 2.62] | 0.87 [0.31, 2.48] | 0.36 [0.11, 1.18] | 0.65 [0.23, 1.81] | 0.1 [0.01, 0.86] | 0.64 [0.17, 2.36] | NA | NA |
| Clinical TNM Stage | 0.05  [0, 0.52] | 0.14 [0.01, 1.61] | 0.3 [0.08, 1.12] | 0.19 [0.05, 0.69] | 0.21 [0.06, 0.77] | 0.22 [0.07, 0.77] | 0.44 [0.12, 1.57] | 1.46 [0.4, 5.29] | 0.39 [0.11, 1.41] | 0.55 [0.17, 1.8] | 0.26 [0.05, 1.07] | 0.46 [0.11, 1.85] | 0.11 [0.02, 0.68] | 0.42 [0.06, 2.81] |
| Pathological Nodal Stage | 0.08 [0.01, 0.74] | 0.19 [0.02, 2.27] | 0.31 [0.09, 1.09] | 0.32 [0.09, 1.08] | 0.25 [0.07, 0.85] | 0.39 [0.12, 1.24] | 0.69 [0.2, 2.33] | 2.78 [0.65, 8] | 0.61 [0.18, 2.08] | 0.69 [0.23, 2.07] | 0.32 [0.07, 1.5] | 0.69 [0.18, 2.71] | 0.15 [0.02, 0.95] | 0.61 [0.09, 3.96] |
|  |  |  |  |  |  |  |  |  |  |  |  |  |  |  |

¶ Odds Ratios and 95% CI was determined using multivariate analysis (logistic regression). Statistical significance was indicated as red. NA, not achieved.

§ WB = salivary rinses collected with an exfoliating brush.

‡ WOB= salivary rinses collected without a brush.

¦ Primary tumor site (Oral cavity) was associated with promoter methylation of *CCNA1*, *DCC*, and *DAPK* in the salivary rinse samples collected with an exfoliating brush, and with promoter methylation of *CCNA1* in the salivary rinse samples collected without brush.
